# *Plasmodium vivax* Malaria during Pregnancy, Bolivia

**DOI:** 10.3201/eid1910.130308

**Published:** 2013-10

**Authors:** Laurent Brutus, José Santalla, Dominique Schneider, Juan Carlos Avila, Philippe Deloron

**Affiliations:** Institut de Recherche pour le Développement, Paris, France (L. Brutus, D. Schneider, P. Deloron);; Université Paris Descartes, Paris (L. Brutus, D. Schneider, P. Deloron);; Ministerio de Salud y Deportes, La Paz, Bolivia (J. Santalla);; Vector-borne Diseases Program, Guayaramerín, Beni, Bolivia (J.C. Avila)

**Keywords:** Plasmodium vivax, parasites, malaria, pregnancy, anemia, birthweight, Bolivia

## Abstract

*Plasmodium vivax* is a major cause of illness in areas with low transmission of malaria in Latin America, Asia, and the Horn of Africa. However, pregnancy-associated malaria remains poorly characterized in such areas. Using a hospital-based survey of women giving birth and an antenatal survey, we assessed the prevalence rates of *Plasmodium* spp. infections in pregnant women in Bolivia, and evaluated the consequences of malaria during pregnancy on the health of mothers and newborns. *P. vivax* infection was detected in 7.9% of pregnant women attending antenatal visits, and placental infection occurred in 2.8% of deliveries; these rates did not vary with parity. Forty-two percent of all *P. vivax* malaria episodes were symptomatic. *P. vivax–*infected pregnant women were frequently anemic (6.5%) and delivered babies of reduced birthweight. *P. vivax* infections during pregnancy are clearly associated with serious adverse outcomes and should be considered in prevention strategies of pregnancy-associated malaria.

In Latin America, where malaria transmission is low and mostly unstable, *Plasmodium vivax* is the most prevalent malaria parasite species. Although ≈3 million pregnant women are exposed to malaria in Latin America each year, the actual number of malaria infections during pregnancy is considerably lower (*1*). Pregnancy-associated malaria is poorly characterized in such areas of low or unstable transmission, as in most areas in which of *P. vivax* is predominant (*2*), but malaria can be severe in all parity groups because most women of childbearing age have low levels of prepregnancy and pregnancy-specific protective immunity to malaria (*3*).

One of the first studies that demonstrated parasitization of the placenta by *P. falciparum* was conducted in Latin America (*4*), and reported serious adverse outcomes, such as miscarriages late in pregnancy or stillbirths. No other study related to pregnancy-associated malaria was conducted in Latin America for ≈80 years until a cohort study investigating *P. vivax* infection during pregnancy in Honduras (*5*) and a case-series report of 143 pregnant women infected with *P. falciparum* in French Guiana (*6*) were reported. Both studies outlined serious adverse outcomes (anemia, preterm delivery, hypotrophy, and stillbirth) associated with malaria by parasite species during pregnancy. More recent studies in the Amazon regions of Brazil and Peru reported increased incidence rates of infection with *P. falciparum*, but not *P. vivax*, in pregnant women (*7*,*8*). Outside Latin America, a few studies reported the effect of pregnancy-associated malaria in unstable malaria settings in Africa and Asia (*9**–**12*), and described increased risks for low birthweight and for maternal anemia as consequences of *P. vivax* infection during pregnancy (*13*,*14*).

Using a hospital-based survey of women giving birth and an antenatal survey, we assessed the prevalence rates of *Plasmodium* infection in pregnant women in 2 malaria-endemic areas of Bolivia. We also evaluated the consequences of malaria infection during pregnancy on the health of mothers and newborns.

## Patients and Methods

### Study Sites

This study was conducted in 2 malaria-endemic areas in Bolivia: the northern district of Guayaramerín in the Amazon region on the border with Brazil, and the district of Bermejo in the southern region on the border with Argentina. In both areas, malaria transmission occurs during the warm and wet season during November–April and is low and unstable; *Anopheles pseudopunctipennis* and *An. darlingi* mosquitoes are the main malaria vectors, respectively (*15*,*16*). *P. vivax* predominates in both areas; *P. falciparum* is present only in Guayaramerín. The annual parasite incidence rates in 2003 were 21.6 and 106.6 infections/1,000 inhabitants in Bermejo and Guayaramerín, respectively. These 2 districts are targeted by routine residual insecticide house-spraying programs that use alphacypermethrin (coverage rate <60%). *P. falciparum* isolates are usually resistant to chloroquine and sulfadoxine/pyrimethamine (*17*), but no chloroquine-resistant *P. vivax* has been reported. Ethical approval for this study was obtained from the Bolivian Ministry of Health (National Institute of Health Laboratories, La Paz).

### Study Population and Data Collection

#### Hospital-based Survey

This survey was conducted during December 2002–August 2004 among women giving birth in 2 district hospitals in which >65% of women in the area give birth. Personal history was obtained for all women, including obstetrical antecedents, place of residence, house insecticide spraying, and signed informed consent. After delivery, a placental blood smear was obtained from the maternal side of the placenta. Thick and thin blood films were prepared. Gestational age of neonates was calculated at birth by using the score of Farr et al. (*18*). Newborns were weighted on a digital scale that was accurate to within 10 g.

#### Antenatal Survey

In Guayaramerín, all consenting pregnant women receiving antenatal care in 2 rural and 5 urban health centers during May 2003–August 2004 were investigated. During each antenatal visit, we performed physical examinations and blood smear examinations for malaria parasites. Giemsa-stained blood smears were read in each center by a trained malariologist. Women with a malaria infection (*P. vivax* or *P. falciparum*) were treated according to the national guidelines at the time (chloroquine or quinine plus clindamycin, respectively). Women were invited to give birth at the district hospital of Guayaramerín and participate in the hospital-based survey.

### Laboratory Studies

Hemoglobin levels were determined by using the cyanomethemoglobin method (HemoCue, Cypress, CA, USA). Peripheral and placental smears were stained with Giemsa, and 200 microscopic fields were examined.

### Definitions

Neonates were classified as premature if they were <37 weeks gestation at birth. Low birthweight was defined as a body weight <2,500 g. Anemia was defined as a hemoglobin level <11 g/dL, and moderate-to-severe anemia as a hemoglobin level <8 g/dL. Asymptomatic malaria infection was defined as the presence of malaria parasites on blood smears in the absence of fever (axillary temperature >37.5°C) or a history of fever in the preceding 48 hours.

### Data Analysis

Twins and stillbirths were excluded from the analysis. *P. vivax* and *P. falciparum* infections were dichotomized independently. Categorical variables were compared by using χ^2^ or Fisher exact tests, and continuous variables were compared buy using the Mann-Whitney test. We used Stata/MP 11 (StataCorp LP, College Station, TX, USA) for multiple linear or logistic regressions (with backward stepwise elimination) to adjust for potential confounding variables (mother’s age, parity, antenatal care attendance, indoor insecticide spraying, site of study, delivery during transmission season, and sex and gestational age of the baby), and to determine the population attributable fraction (PAF), which is also known as the etiologic fraction, or that proportion of all events (severe anemia, low birthweight) associated with the factor of interest (e.g., *P. vivax* or *P. falciparum* infection).

## Results

### Hospital-based Survey

During December 2002–August 2004, a total of 1,003 women in Guayaramerín and 504 women in Bermejo had singleton births at the 2 district hospitals. In both hospitals, mean parity and proportion of primiparous women were similar (Table 1). However, women were younger (mean ± SD age 23.2 ± 6.4 years vs. 24.2 ± 6.7 years; p = 0.008) and had more antenatal visits (4.7 ± 2.0 visits vs. 3.7 ± 2.1 visits; p<0.001) in Guayaramerín than in Bermejo. The proportion of women without any antenatal visit was 4 times higher (7.8% vs. 1.9%; p<0.001) in Bermejo than in Guayaramerín. Women lived less often in rural settlements (6.8% vs. 22.7%; p<0.001) and had babies more often during the transmission season (60.0% vs. 52.6%; p = 0.006) in Guayaramerín than in Bermejo. Rates of low-birthweight and moderate-to-severe maternal anemia at birth were similar in both places.

**Table 1:** Baseline characteristics of women and babies at delivery during a hospital-based survey, Guayaramerín and Bermejo Bolivia, 2002–2004*

Characteristic	Guayaramerín, n = 1,003	Bermejo, n = 504	p value
Mothers			
Age, y	23.2 ± 6.4	24.2 ± 6.7	**0.008**
No. previous pregnancies	2.1 ± 2.4	1.9 ± 2.1	0.40
Primiparae	31.7	28.4	0.18
No. antenatal visits	4.7 ± 2.0	3.7 ± 2.1	**<0.001**
No antenatal visit	1.9	7.8	**<0.001**
Houses with indoor insecticide spraying	55.0	50.3	0.098
Women living in rural settlements	6.8	22.7	**<0.001**
Delivery during transmission season	60.0	52.6	**0.006**
Hemoglobin level, g/dL	11.1 ± 2.0	11.4 ± 1.9	**<0.001**
Moderate-to-severe anemia, hemoglobin level <8 g/dL	6.8	6.3	0.70
Babies			
Girls	47.5	49.8	0.41
Birthweight, g	3,310 ± 509	3,383 ± 515	**0.003**
Low birthweight, <2,500 g	5.0	4.8	0.83
Premature babies, <37 weeks	7.6	4.4	**0.018**
Placental *Plasmodium vivax* infection	2.7	3.0	0.73
Placental *P. falciparum* infection	0.4	NA	NA

*Values are mean ± SD or percentage. Significant values (p<0.05) are indicated in **boldface**. NA, not applicable.

Among 967 women who had babies in Guayaramerín and had a placental smear, 26 (2.7%) were had *P. vivax* infections in placental blood. In addition, 4 (0.4%) had placental *P. falciparum* infections. Among 500 women who had babies in Bermejo and had a placental examination, 15 (3.0%) had *P. vivax* infections in placental blood. Because of these differences, we further adjusted for study area to evaluate the effects of *P. vivax* infection in pregnant women. We further distinguished infections by *P. falciparum* or *P. vivax* for the analysis.

The risk for placental *P. vivax* infection increased during the transmission season in both places (adjusted odds ratio [OR] 2.7, 95% CI 1.3–5.6, p<0.007). There was no effect of parity, mother’s age, antenatal care attendance, or indoor insecticide spraying on placental *P. vivax* prevalence in both districts.

Women with placental *P. vivax* infections were more likely than noninfected mothers to have a low-birthweight baby (OR adjusted for study site 3.6, 95% CI 1.4–8.9) (Table 2). These women were also more likely than noninfected women to have moderate-to-severe anemia (adjusted OR 2.5, 95% CI 1.0−6.2).

**Table 2:** Risks for low birthweight and maternal anemia associated with placental *Plasmodium vivax* infections, Guayaramerín and Bermejo, Bolivia, 2002–2004*

Risk	OR for adverse condition (95% CI), p value		Adjusted OR† (95% CI), p value
	Bermejo	Guayaramerín	
Low-birthweight babies of women with or without placental infection	3.4 (0.7–16.1), 0.10	3.7 (1.2–11.2), **0.01**	**3.6 (1.4–8.9), 0.003**
Moderate-to-severe anemia among women with or without placental infection	4.3 (1.1–16.4), **0.02**	1.8 (0.5–6.1) 0.35	**2.5 (1.0–6.2), 0.03**

*OR, odds ratio. Significant values (p<0.05) are indicated in **boldface**. †Adjusted OR after stratifying for study sites.

Factors associated with mean birthweight in a multiple linear regression model are shown in Table 3. Mean birthweight was reduced in premature (−752 g), female (−151 g), first-born (−168 g), and second-born babies (−79 g), as well as in babies born to mothers living in Guayaramerín (−52 g), women who had no antenatal visits (−112 g), and women with placental *P. vivax* infections (−181 g). Mean birthweight was increased in babies born to women >35 years of age (+181 g) or women 25–35 years of age (+102 g). Logistic regression (Table 3) showed that preterm delivery (p<0.001) and placental *P. vivax* infections (OR 6.2, 95% CI 2.2–17.6, p<0.001) were associated with an increased risk for low-birthweight babies. The population attributable risk for low-birthweight babies associated with *P. vivax* infection was 6.1% (95% CI 0.4%–11.4%).

**Table 3:** Factors associated with mean birthweight and risk for low birthweight babies, excluding *Plasmodium falciparum* infections, during hospital-based survey, Guayaramerín and Bermejo, Bolivia, 2002–2004*

Characteristic	Multiple linear regression, n = 1,417		Mutlivariate logistic regression, n = 1,417	
	Adjusted difference in mean birthweight, g (95% CI)†	p value	Adjusted OR for low birthweight (95% CI)	p value
Baby				
Mature	0	NS	1	NS
Premature	–752 (−849 to −656)	**<0.001**	37.8 (20.9–68.3)	**<0.001**
Boy	0	NS	1	NS
Girl	–151 (−198 to −105)	**<0.001**	1.7 (0.98–3.1)	0.06
Mother				
Multiparous	0	NS	1	NS
Secondiparous	–79 (−146 to −13)	**0.02**	1.6 (0.91–2.9)‡	0.10
Primiparous	–168 (−232 to −103)	**<0.001**	NS	NS
<25 y of age	0	NS	NS	0.56
25–35 y of age	102 (38–167)	**0.002**	NS	NS
>35 y of age	181 (83–279)	**<0.001**	NS	NS
Antenatal visit	0	NS	NS	0.22
No antenatal visit	–112 (−235 to 10)	0.07	NS	NS
Bermejo	0	NS	NS	0.47
Guayaramerín	–52 (−102 to −2)	**0.04**	NS	NS
Noninfected placenta	0	NS	1	NS
*P. vivax–*infected placenta	–181 (−321 to −41)	**0.01**	6.2 (2.2–17.6)	**<0.001**

*OR, odds ratio; NS, not significant. Significant values (<0.05) are indicated in **boldface**. Multivariate models adjusting for mother’s age, number of previous pregnancies, antenatal visits, houses with indoor insecticide spraying, site of study, delivery during transmission season, mother’s anemia, and sex and gestational age of the baby. Only significant variables (p<0.10) from linear regression model are shown. The same variables were used for the logistic regression model. †Baseline mean birthweight was 3,537 g. ‡First and second pregnancies combined compared with multiparous women.

We used a multiple linear regression model to identify factors associated with changes in mean hemoglobin levels (Table 4). Mean hemoglobin level was significantly reduced in multiparous women (−0.28 g/dL; p = 0.012), women in Guayaramerín (−0.38 g/dL; p = 0.001), and women with placental *P. vivax* infections (−0.70 g/dL; p = 0.026). Logistic regression showed that placental *P. vivax* infection remained independently associated with an increased risk for moderate-to-severe anemia (OR 2.5, 95% CI 1.04–6.2, p = 0.04). The population attributable risk for moderate-to-severe maternal anemia associated with *P. vivax* infection was 3.5% (95% CI –1.2% to 8.1%).

**Table 4:** Factors associated with mean hemoglobin level and risk for moderate-to-severe anemia, excluding *Plasmodium falciparum* infections, during hospital-based survey, Guayaramerín and Bermejo, Bolivia, 2002–2004*

Characteristic	Multiple linear regression, n = 1,439		Logistic regression, n = 1,439	
	Adjusted difference in mean hemoglobin level, g/dL (95% CI)†	p value	Adjusted OR for moderate-to-severe anemia (95% CI)	p value
Primiparous mother	0	NS	NS	0.23
Multiparous mother	–0.28 (−0.51 to −0.06)	**0.012**	NS	NS
Bermejo	0	NS	NS	0.42
Guayaramerín	–0.38 (−0.59 to −0.16)	**0.001**	NS	NS
Noninfected placenta	0	NS	1	NS
*P. vivax–* infected placenta	–0.70 (−1.32 to −0.09)	**0.026**	2.5 (1.04–6.2)	0.04

*OR, odds ratio; NS, not significant. Significant values (<0.05) are indicated in **boldface**. Multivariate models adjusting for number of previous pregnancies, antenatal visits, houses with indoor insecticide spraying, site of study, and delivery during transmission season. Only significant variables (p<0.10) from the linear regression model are shown. The same variables were used for the logistic regression model. †Baseline mean hemoglobin level was 11.7 g/dL.

In contrast to placental *P. vivax* infection, placental *P. falciparum* infection was more likely to occur in primiparous women than in multiparous women (0.7% vs. 0.1%; p = 0.05). After exclusion of Bermejo and *P. vivax* infections, the risk for low birthweight increased in premature babies (OR 31.2, 95% CI 15.7–62.1; p < 0.001) and in babies born to mothers with placental *P. falciparum* infections (OR 5.1, 95% CI 1.6–16.6; p = 0.006).

### Antenatal Survey

During May 2003–August 2004, a total of 359 women had antenatal visits and subsequently gave birth in Guayaramerín. Mean ± SD parity was 1.9 ± 2.2 (range 0–13), mean ± SD age was 22.8 ± 6.2 years (range 13–45 years), mean ± SD number of antenatal visits was 4.9 ± 1.8 (range 1–10), and mean ± SD number of blood screenings was 3.4 ± 1.9 (range 1–9). Of these women, 330 had no documented malaria episodes, 1 was infected with *P. falciparum*, and 28 (7.8%; 95% CI 5.0–10.6) had ≥1 *P. vivax* infection between first antenatal visit and delivery (20 women had 1 infection, 6 had 2 infections, and 2 had 3 infections). Of these 28 women, 57.5% had febrile illness and 42.5% were asymptomatic. A total of 143 women (42.8%; 95% CI 37.5%–48.1%) had anemia and 23 (6.9%; 95% CI 4.1–9.6) had moderate-to-severe anemia. Fourteen women (3.9%, 95% CI 1.9%–5.9%) gave birth to low-birthweight babies. The proportion of women with *P. vivax* infections was similar in primiparae (7.3%) and multiparae (8.1%). The *P. vivax* infection rate was 4.3% (3/69), 4.6% (10/215), and 6.5% (22/336) during the first, second, and third trimesters, respectively.

After logistic regression, the risk for moderate-to-severe anemia at delivery remained associated with parity and was higher in multiparae than in primiparae (OR 3.9, 95% CI 1.1–13.6; p = 0.03) and in women with *P. vivax* infection during antenatal visits (OR 3.7, 95% CI 1.2–11.1; p = 0.02). The proportion of low-birthweight babies was higher in women who had been infected with *P. vivax* during pregnancy (17.9%) than in noninfected women (2.7%; p<0.001). The odds of low-birthweight babies born to mothers without *P. vivax* infection, with 1 infection, and with ≥2 infections during the antenatal survey were 2.8%, 17.6%, and 33.3%, respectively (p<0.001, by score test for trend of odds). The mean birthweight of babies born to women who had been infected with *P. vivax* during pregnancy was 289 g lower than that of babies born to noninfected mothers (mean ± SD 3,054 ± 535 g vs. 3,343 ± 480 g; p = 0.008). The mean hemoglobin level for women who were infected with *P. vivax* during pregnancy was 0.74 g/dL lower than that for noninfected mothers (mean ± SD 10.3 ± 1.9 g/dL vs. 11.0 ± 2.1 g; p = 0.06).

Factors associated with a change in mean birthweight by a multiple linear regression model are shown in Table 5. Mean birthweight was lower in girls (−135 g), premature babies (−426 g), and first-pregnancy babies (−181 g), as well as in babies born to anemic mothers (−92 g) or to mothers infected with *P. vivax* during pregnancy (−266 g). Logistic regression showed that preterm delivery (p = 0.001) and *P. vivax* infection during pregnancy (OR = 8.8, 95% CI 2.4–32.5) were associated with low birthweight.

**Table 5:** Factors associated with mean birthweight and risk for low birthweight during antenatal survey, Guayaramerín and Bermejo, Bolivia, 2003–2004*

Characteristic	Multiple linear regression, n = 329		Logistic regression, n = 329	
	Adjusted difference in mean birthweight, g (95% CI)†	p value	Adjusted OR for low birthweight (95% CI)	p value
Baby				
Mature	0	**<0.001**	1	NS
Premature	–426 (−626 to −227)	NS	10.5 (2.8–39.8)	**0.001**
Boy	0	NS	NS	0.98
Girl	–135 (−237 to −34)	**0.009**	NS	0.98
Multiparous mother	0	NS	NS	0.52
Primiparous mother	–181 (−287 to −75)	**0.001**	NS	0.52
No anemia at delivery	0	**0.08**	NS	0.69
Anemia at delivery	– 92 (−195 to 10)	0.08	NS	0.69
Not infected at antenatal visits	0	NS	1	NS
Infected with *Plasmodium vivax* at antenatal visits	– 266 (−453 to −78)	**0.006**	8.8 (2.4–32.5)	**0.001**

*OR, odds ratio; NS, not significant. Significant values (<0.05) are indicated in **boldface**. Multivariate models adjusting for mother’s age, maternal anemia, number of previous pregnancies, and sex and gestational age of the baby. Only significant variables (p<0.10) from the linear regression model are shown. The same variables were used for logistic regression model. †Baseline mean birthweight was 3,538 g.

## Discussion

Long after the first studies on pregnancy-associated malaria conducted in Africa, most studies in Latin America during the past decade, mainly case-series studies, reported numerous adverse conditions, such as a high frequency of maternal anemia, miscarriage, stillbirth, preterm delivery, and low birthweight (*6*,*19**–**23*), related to malaria infections with *P. falciparum* or *P. vivax* during pregnancy. As observed in other malaria-endemic areas, a cohort study in Peru and a cross-sectional study in Brazil reported a 2.5-fold increase in susceptibility to *P. falciparum* malaria among pregnant women than among nonpregnant women (*7*,*8*). Neither study demonstrated a similar higher frequency of *P. vivax* infection in pregnant women.

In the current study, *P. vivax* infection was detected in 7.9% of pregnant women attending antenatal visits. This proportion is similar to rates in other settings, such as in Thailand (6.4%–8.5%) and Honduras (9.1%) (*5**,**9**,**13*). These findings suggest a constant proportion of *P. vivax* infections during pregnancy in different malaria transmission patterns. In Thailand, 23% of all *P. vivax* malaria episodes were symptomatic (*13*), but this rate reached 42% in Bolivia. In addition to possible differences in background immunity resulting from more unstable transmission in Bolivia, this difference might also be caused by prompt diagnosis and treatment on a weekly basis in the study in Thailand, which enabled parasite detection and cure before onset of symptoms. In our study, diagnosis and treatment were performed monthly, which is the approximate interval between 2 antenatal visits, which enabled a longer time for symptoms to develop.

Among pregnant women, primiparae women are most vulnerable to *P. falciparum* infections, and the difference between primiparae and multiparae women is more pronounced in areas of stable than unstable malaria transmission (*11*,*12*,*24*). We observed similar differences, despite a limited number of *P. falciparum–*infected women. In contrast and consistent with previous reports (*10*), *P. vivax* infection was observed in a similar proportion of women of all parities. However, 1 study reported an increased risk for *P. vivax* infection in primiparae than in multiparae (*13*).

A high proportion of pregnant women in both study sites in Bolivia had anemia, and the proportion of women with moderate-to-severe anemia increased with parity. As observed in unstable malaria transmission settings, the risk for maternal anemia was more pronounced in multiparae than in primiparae women (*9*,*10*,*12*). In our study, *P. vivax* infection was associated with a reduction of 0.7 g/dL in the hemoglobin level of infected pregnant women than that of noninfected women. A similar difference (0.8 g/dL) was observed in Honduras (*5*) between *P. vivax–*infected and noninfected women. Logistic regression showed that the risk for maternal anemia was associated with *P. vivax* infection at delivery, multiparity, and the study district in northern Bolivia. In our antenatal cohort study, *P. vivax* infection acquired during pregnancy remained independently associated with the risk for moderate-to-severe anemia. A similar relationship was observed in Thailand (*13*). Other studies also reported the effect of infection with *P. falciparum* or *P. vivax* during pregnancy on the risk for maternal anemia, but confounding malaria species could have led to classification bias (*9*,*10*,*12*,*19*).

Babies born to *P. vivax–*infected mothers showed a major mean birthweight reduction of 181 g when compared with babies born to noninfected women, which is consistent with observations in Thailand and Honduras (107 and 198 g, respectively) (*5*,*13*). Mean birthweight was also highly reduced in case of preterm delivery, of poor antenatal clinic attendance, and in babies born to first- and second-pregnancy babies. These factors were consistently identified in studies performed in unstable malaria settings (*10*,*13*). In our study, placental *P. vivax* infection was associated with a 6-fold higher risk for low birthweight, which is ≈4 times higher than the risk estimated in Thailand (*13*). However, in Madagascar, in areas of unstable malaria transmission, the risk for low birthweight associated with *P. falciparum* infection was 2.5 times that in areas with stable transmission (*11*). As suggested by the higher proportion of symptomatic infections in our study in Bolivia, the index of stability may be lower in Bolivia than in Asia if one takes into account a higher risk for low birthweight associated with *P. vivax* infection. In our study, the risk for low birthweight increased with the number of *P. vivax* infections that occurred during pregnancy (by test for trend). These data are consistent with a similar dose-dependent effect in a study in Thailand, which reported a greater reduction in birthweight in mothers infected ≥5 times than in mothers infected only 1 time (*9*).

In Bolivia, the PAF of moderate-to-severe anemia associated with *P. vivax* malaria was 3.5%, and the PAF for low birthweight was 6.1% for *P. vivax*. Our estimation is consistent with the 2%–15% estimation of the PAF for severe anemia related to *P. falciparum* in malaria-endemic areas (*25*). In contrast, *P. vivax* seemed to have less of an effect on the risk for low birthweight than *P. falciparum* in malaria-endemic areas in Africa (risk 8%–14% estimated by Steketee et al.) (*25*).

Our cross-sectional survey has limitations, including selection biases (if most women do not attend selected structures or because they give birth at home because private and nongovernment organization sectors predominate in the public sector) and representativeness. In the 2 districts we studied, private and nongovernment organization sectors are negligible and most births are in public sector facilities. However, ≈25%–33% of births were at home in the regions studied. This factor is a possible limitation because we did not assess deliveries at home. This limitation is similar for prenatal visits, but the number of pregnant women who receive prenatal care in Bolivia is high (>80%). We conducted the prenatal follow-up study in 7 health centers to ensure representativeness. To avoid missing the transmission season, we conducted the study in >1 calendar year.

Although *P. vivax* infections are clearly associated with serious adverse outcomes during pregnancy, accumulation of *P. vivax* in the placenta has not been reported. *P. vivax–*infected erythrocytes can bind chondroitin sulfate A, the placental binding receptor (*26*), but at a 10-fold lower level than *P. falciparum–*infected erythrocytes (*27*), and at a similar level in isolates from pregnant women or nonpregnant persons (*28*). The paucity of *P. vivax* in the placenta has been reported (*29*,*30*), and *P. vivax* has been inconsistently associated with the presence of malaria pigment in the placenta, but not associated with placental pathologic changes (*14*,*31*). High circulating levels of inflammatory cytokines during the paroxysms of *P. vivax* malaria (*32*) may be sufficient to impair fetal growth and cause maternal anemia, as hypothesized by Nosten et al. (*2*). Moreover, rosette formation is a frequent cytoadhesive phenotype in *P. vivax* infections and has been associated with an increased risk for anemia (*28*). Nevertheless, phenomena involved in pathologic mechanisms specific for *P. vivax* infection during pregnancy remain to be elucidated.

*P. falciparum* and *P. vivax* infections during early pregnancy have been shown to result in impaired fetal growth (*33*), which emphasizes the need to include early pregnancy in the prevention strategies of pregnancy-associated malaria. In addition, almost half of *P. vivax* infections were asymptomatic, suggesting that women should be screened for malaria at every antenatal clinic visit, and treated if test results were positive. Although the effects of *P. vivax* infection during pregnancy have become increasingly documented, health personnel in malaria-endemic areas of Latin America still largely ignore recommendations for diagnosis and treatment of malaria in pregnant women (*34*). Efforts should be undertaken to increase staff training to limit the effect of malaria during pregnancy.
